# Children and the Cinema

**Published:** 1920-10-16

**Authors:** 


### CHILDREN AND THE CINEMA.

The remarkably rapid spread of the cinema craze and the fact, as evidenced by widespread building operations and the activities of company promoters, that its height has by no means been reached, brings forward a variety of problems both medical and social. For the moment it is no part of our intention to discuss the latter; they have already been almost over-discussed in the daily press, and are concerned with the type of picture rather than the theatre itself. But the various medical ills which have been meanwhile attributed to the children's very natural love of the "movie shows" may well claim our attention for a moment. (See also p. 55.)

The most obvious and most frequent question arises with the supposed damage to the children's eyes, and we have little hesitation in saying that the many popular estimates of this danger have been greatly exaggerated. In the first place, definition of the proper conditions, particularly for young eyes, under which pictures could be exhibited were laid down by the Cinema Commission which sat in 1917. Also, generally speaking, there is considerable evidence that the cinematograph as now seen does no harm whatever to the eyes of young or old, and we believe that there is no instance of ocular harm occurring among those whose professional duties require their close observance of films, daily for many hours at a stretch. Close proximity to the screen may have its disadvantages, and there the cheapest seats, and therefore the children are generally found; but even this sinks into insignificance compared with the ordinary hygienic risks associated with any influence which tends closely to congregate young children. The problem of efficient ventilation is of far greater importance than the risk of eye strain, and nowadays it must in fairness be said that, of all places where one meets our juveniles collected, the cinema is in by no means invariable possession of the closest atmosphere. Rigid requirement of a high ventilation standard is essential, but daily we see such conditions improve, and viewing the situation from the standpoint of purely medical hygiene it is difficult to agree that the picture theatre of the future will have any directly damaging influence on the nation's children. Instead we see the possibility that cinemas may and should become a potent factor in health education of old as well as young, and when this power is utilised to the full, the indirect hygienic benefit should many times outweigh the direct bodily harm, which, in any case, exists for the most part in the critic's imagination alone.

We realise that the cinema has, even now, restricted use for juvenile education in this country, but in England such developments are, and, we fear, will always be, very slow. On the other hand, in America the extraordinary vividness with which almost every known explanation of Nature's laws can be brought home to either the youthful or the adult mind is utilised to the full. While we are still debating upon this educational "problem" we have absolutely at hand proved experience in a method in use for years past in the States. There even the Churches use this mechanical adjunct to their educational efforts, and we see it estimated that in a very few years some eight per cent. of their churches will have a cinema in regular use.

A few months ago we had opportunity to express genuine approval of some at least of the films which were produced with the object of providing enlightenment on the medico-social facts of venereal disease. Undoubtedly some of these productions have achieved good results, and the fact that they have represented attempts upon one of the most difficult educational problems of the day makes it only more obvious that truly remarkable achievements might be expected from a campaign along a path of far less subtle resistance—namely, the hygienic instruction of our children. We do not suggest "movie shows" devoted entirely to such subjects, but simply the insertion of short, vividly educational films in the midst of the ordinary programmes. It cannot be expected that purely educational exhibitions will attract a persistently big house, but to the general cinema programme the children will continue to crowd, and there, in the existing palaces, lies the opportunity to instruct them without suggestion of the irksomeness habitually associated with "lessons."

